# Teachers’ experiences of delivering youth vaping prevention materials in schools in England and Scotland: A cross-sectional online survey

**DOI:** 10.1371/journal.pone.0335474

**Published:** 2025-11-07

**Authors:** Ashley Lee, Hannah Walsh, Matilda Nottage, Stephanie Fincham-Campbell, Eve Taylor, Deborah Robson, Mark Conner, Katherine East

**Affiliations:** 1 Evidence to Impact, Bristol,, England; 2 National Addiction Centre, Institute of Psychiatry, Psychology and Neuroscience, King’s College London, London,, England; 3 Department of Behavioural Science and Health, University College London, London, England; 4 School of Psychology, University of Leeds, Leeds, England; 5 Department of Primary Care & Public Health, Brighton and Sussex Medical School, University of Brighton and University of Sussex, Brighton, England; Dublin City University, IRELAND

## Abstract

INTENT is an evidence-based smoking prevention programme for secondary school pupils in Great Britain (GB) that was recently expanded to include vaping information. Little research has evaluated GB-specific youth vaping prevention materials. This study assesses teachers’ experiences of delivering INTENT’s vaping prevention materials in England and Scotland. Teachers who delivered INTENT in England/Scotland (N = 45) were surveyed online in 2024, investigating their i) characteristics, ii) experiences of delivering INTENT, iii) perceived impact of INTENT on their pupils, and iv) perceived levels of smoking and vaping in their school. Teachers frequently reported finding pupils possessing/using vapes (51% at least once a week) and perceiving vaping as a problem (96%) in their school, more than cigarettes (4%, 35%, respectively). Teachers had positive or somewhat positive experiences delivering the INTENT vaping prevention materials (96%), perceived that pupils were engaged either ‘a lot’ or ‘somewhat’ (98%), and felt the materials encouraged pupils to make informed choices about vaping (89%). While most teachers reported a change in vaping harm perceptions after INTENT (82%), misperceptions that vaping is equally/more harmful than smoking remained high (65%). A third of teachers did not perceive a change in pupils’ vaping (35.6%) or smoking (26.7%), or did not know (31.1%, 48.9%, respectively). In conclusion, this study found that INTENT shows potential to improve teachers’ knowledge about vaping and smoking and to challenge vaping harm perceptions, and that experiences with delivery and student engagement were positive. Studies evaluating the impact of INTENT and other school-based interventions on school pupils’ vaping and smoking perceptions and behaviours are required.

## Introduction

Vaping (e-cigarette use) has increased among youth in Great Britain over the past decade [1]. Between 2013 and 2025, the prevalence of current vaping among youth aged 11–17 rose from 1% to 7% [1]. Vaping is substantially less harmful to health than smoking and can help people to quit smoking [2–6]. Most e-cigarettes contain nicotine, which has addictive potential, particularly when delivered rapidly through inhalation [3,4]. Biomarker studies among youth indicate that those who vape have nicotine exposure at levels similar to their peers who smoke tobacco [7]. Vaping also exposes young people to potentially harmful toxicants at lower levels than smoking but higher than non-use [8], and the long-term health effects of these exposures remain uncertain. For youth, both nicotine dependence and toxicant exposure are important risks, as early initiation can sustain nicotine use and extend toxicant exposure across the lifespan [9]. It is also illegal for retailers to sell vaping products to under-18s in Great Britain [10]. Youth who do not smoke should therefore be discouraged from vaping.

Evidence suggests that youth vaping prevention campaigns, including those delivered in school settings, can increase perceptions that vaping can harm health and deter youth from trying vaping [3,11–13]. Most campaigns focus on the absolute risks of vaping (i.e., that vaping risks addiction and harm to health) and some contain statements that vaping causes lung injuries, heart and respiratory diseases, and cancers [3,11–15]. Few campaigns that have been evaluated provide nuanced information about both absolute and relative harms (i.e., that vaping is less harmful than smoking but carries some risks) [3,11,13]. While campaigns focussing on the absolute risks of vaping may reduce vaping, they can also increase misperceptions that vaping is equally or more harmful than smoking [3,13,16]. This could, in turn, increase the likelihood of smoking (a more harmful behaviour) or decrease the likelihood of using a vape in a smoking cessation attempt [3,13,16,17]. Vaping prevention campaigns must therefore be carefully designed to ensure that they do not inadvertently increase misperceptions and encourage smoking. Most evidence on youth vaping prevention campaigns to date is from the US and Canada, with little research from Great Britain [11–13]. In 2022, 25% of youth in England reported being exposed to vaping campaigns or messages in schools [18].

INTENT is an evidence-based smoking prevention programme targeted at secondary school pupils in Great Britain, developed over a period of 20 years and recently expanded to include information about vaping. INTENT is based on implementation intentions, which are specific “if-then” plans on how, where, and when to perform a behaviour (e.g., “If I am offered an e-cigarette at the bus stop, then I will say ‘no thank you, it gives me a dry mouth’”) [19]. The effects of forming implementation intentions are often contingent on the presence of strong motivation or goal intention to perform the behaviour [20,21]. Interventions involving implementation intentions therefore often include information about the risks and/or benefits of engaging in a behaviour [22,23]. Specifically, INTENT provides information about the risks of smoking and vaping.

INTENT reduced youth smoking initiation from 36% to 29% in a pragmatic Randomised Controlled Trial (RCT) [22] and was subsequently deployed in 2021 across secondary schools in England and Scotland to tackle smoking initiation. INTENT was also found to significantly weaken the association between vaping and subsequent smoking among school pupils [24]. In 2022, following concerns about youth vaping among local authorities and teachers and the lack of evidence-based educational vaping materials in Great Britain, INTENT was expanded to include vaping-specific implementation intentions and health risk information. INTENT’s vaping prevention materials have not yet been evaluated.

Understanding teachers’ experiences and perceptions of vaping and of delivering vaping prevention materials, such as those used within INTENT, is important for several reasons. First, schools represent a key context for promoting health behaviours, with implications across the lifespan [25,26]. Second, improving teachers’ knowledge about health behaviours can have wide-ranging impact [26], particularly because teachers educate numerous young people each year (e.g., on average, there are 18 school pupils per teacher in England) [27]. Evidence also suggests that school-based, teacher-led interventions for changing health behaviours are feasible and cost-effective [22]. Third, teachers in Great Britain have reported concerns over what to tell children about vaping due to mixed messaging and absence of official guidance, and they often have misperceptions themselves about the harms of vaping relative to smoking [28] that could inadvertently encourage smoking over vaping [18]. Fourth, intervention success may be partly dependent on delivery, so it is important to ensure delivery experiences are positive. Studies among teachers in the US suggest that there is considerable variation in school approaches to youth vaping and that many teachers feel underprepared to address pupils’ tobacco and nicotine use [15,29].

This project therefore aimed to assess teachers’ experiences with delivering the INTENT youth vaping prevention materials to school pupils in England and Scotland. To our knowledge this is the first study to assess teachers’ perceptions of vaping and vaping prevention materials outside of the US [14,15,29].

## Methods

### Development and delivery of INTENT youth vaping prevention materials

The INTENT youth vaping prevention materials were developed in collaboration with INTENT’s customers (e.g., local authorities) and university researchers (University of Leeds, King’s College London) between October 2022 and August 2023. The Office of Health Improvement and Disparities (OHID), a government office that is dedicated to improving the nation’s health and has commissioned vaping evidence reviews [3], also provided feedback on the materials. Teachers were asked to deliver the vaping materials after the smoking prevention programme, which focussed on the harms of smoking and implementation intentions to avoid smoking initiation.

**Fig 1:**
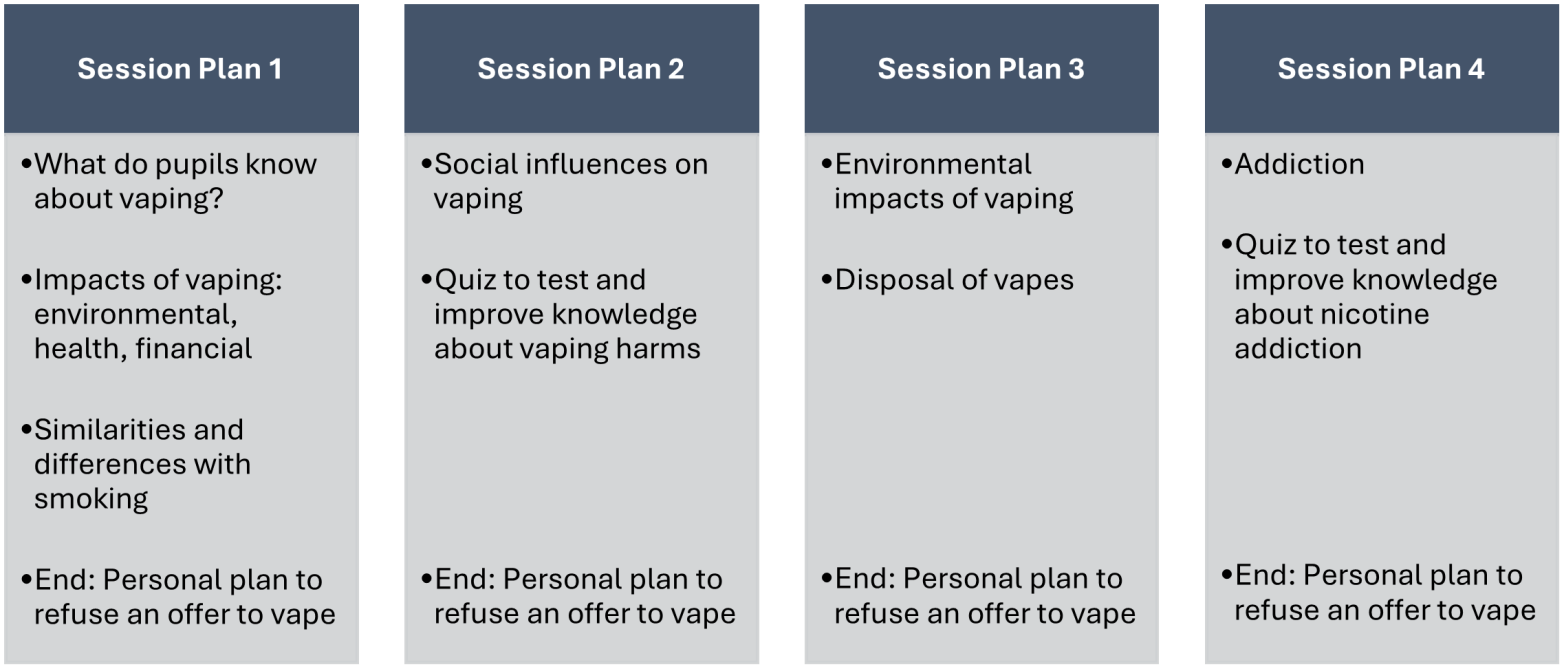
Overview of the INTENT vaping prevention materials session plans.

The INTENT vaping prevention materials comprised four session plans (Fig 1). Briefly, session plans covered what pupils know about vaping, the impacts of vaping, and to compare vaping to smoking (Session Plan 1), social influences on health behaviours including vaping, including a quiz on harm perceptions and smoking cessation (Session Plan 2), the environmental impacts of vaping (Session Plan 3), and addiction (in general, and specific to nicotine; Session Plan 4). Pupils were also asked at the end of each session to create a personal plan to refuse an offer to vape. The session plans are described in detail in the Supplementary Materials. Further details of the intervention are available upon reasonable request from Evidence to Impact [30].

The INTENT youth vaping prevention materials are designed to be delivered between Year 7 in England (Secondary 1 (S1) in Scotland; typically aged 11–12 years) to Year 10 (S4 in Scotland; typically aged 14–15 years). The recommendation is that between 40 and 50 minutes are allocated for each session and that they are incorporated into the curriculum during Personal, Social, Health, and Economic Education (PSHE) lessons in England or Curriculum for Excellence (CfE) lessons in Scotland.

## Design

This was a cross-sectional study in which teachers were asked to complete a 10-minute online survey administered through Qualtrics. Data were collected between 15 February and 31 July 2024. Inclusion criteria were that teachers had to have delivered at least Session Plans 1 and 2 of INTENT, including the quiz in Session Plan 2, because these sessions contained the most information about the health harms of vaping. The Kings College London ethical review committee approved the study (REF: MRA-23/24–39974).

### Sample and recruitment

All 8 organisations covering 18 local authorities in England and Scotland that commissioned INTENT were approached and their consent was requested to approach schools in their areas (131 schools total). Of these 8 commissioning organisations, 6 provided consent to approach schools in their areas (119 schools; see Table S1 in S1 File). Teachers in the schools were then approached for recruitment via email. Participants provided informed written consent as part of an online survey during data collection. Teachers that completed the survey received a £10 online retail voucher upon successful completion of the study. A total of 70 teachers from 10 schools were recruited, of whom 25 did not meet inclusion criteria or did not complete the survey, leaving 45 teachers in the analytic sample. Of the 45 included, 26 teachers delivered all 4 Session Plans. Table S2 (S1 File) shows the characteristics of the 45 teachers who were included in the sample against the 25 who were not included; there were few notable differences.

## Measures

Measures and response options are shown in Tables 1–3. The full survey is available online osf.io/4rtav [31].

**Table 1:** Teacher characteristics (N = 45).

	N	%
Role within school		
Head of Department	4	8.9
Teacher	28	62.2
Teaching Assistant	1	2.2
Other (e.g., Assistant Principal, Progress Leader, Special Education Needs Coordinator)	11	24.4
Prefer not to say	1	2.2
Age group		
25 - 34	22	48.9
35 - 44	11	24.4
45 - 54	11	24.4
Prefer not to say	1	2.2
Gender		
Man	17	37.8
Woman	27	60.0
Prefer not to say	1	2.2
E-cigarette use (vaping)		
I currently use them daily	1	2.2
I currently use them weekly but not daily	1	2.2
I currently use them monthly but not weekly	0	0.0
I have tried them but do not use them monthly	13	28.9
I have never tried them	29	64.4
Don’t know	1	2.2
Cigarette use (smoking)		
I currently use them daily	1	2.2
I currently use them weekly but not daily	1	2.2
I currently use them monthly but not weekly	0	0.0
I have tried them but do not use them monthly	21	46.7
I have never tried them	21	46.7
Don’t know	1	2.2
How frequently do you find pupils in your school in possession of, or using, e-cigarettes?		
Daily	9	20.0
Multiple times a week	10	22.2
About once a week	4	8.9
Fortnightly	2	4.4
Monthly	5	11.1
Less often than monthly	9	20.0
Never	2	4.4
Don’t know	4	8.9
How frequently do you find pupils in your school in possession of, or using, tobacco cigarettes?		
Daily	0	0.0
Multiple times a week	1	2.2
About once a week	1	2.2
Fortnightly	1	2.2
Monthly	1	2.2
Less often than monthly	14	31.1
Never	15	33.3
Don’t know	12	26.7
Do you think vaping e-cigarettes among pupils in your school is a problem?		
Yes, a lot	28	62.2
Yes, a little	15	33.3
Not at all	1	2.2
Don’t know	1	2.2
Do you think smoking cigarettes among pupils in your school is a problem?		
Yes, a lot	1	2.2
Yes, a little	15	33.3
Not at all	12	26.7
Don’t know	17	37.8

**Table 2:** Teachers’ experiences with INTENT (N = 45).

	N	%
How would you rate your overall experience of delivering the INTENT vaping prevention materials?		
Very positive	18	40.0
Somewhat positive	25	55.6
Neither	1	2.2
Somewhat negative	1	2.2
Very negative	0	0.0
Don’t know	0	0.0
How would you rate the content of the INTENT vaping prevention materials?		
Liked the content	17	37.8
Neither liked nor disliked the content	24	53.3
Disliked the content	4	8.9
Don’t know	0	0.0
Did you adhere to the session plan(s)?		
All of the time	22	48.9
Most/some of the time	19	42.2
Not at all	4	8.9
Don’t know	0	0.0
How easy/difficult was it to prepare for delivering the INTENT vaping prevention materials?		
Very easy	23	51.1
Somewhat easy	16	35.6
Neither easy nor difficult	6	13.3
Somewhat difficult	0	0.0
Very difficult	0	0.0
Did not prepare	0	0.0
Don’t know	0	0.0
How easy/difficult was it to deliver the INTENT vaping prevention materials?		
Very easy	20	44.4
Somewhat easy	22	48.9
Neither easy nor difficult	3	6.7
Somewhat difficult	0	0.0
Very difficult	0	0.0
Don’t know	0	0.0
Reasons for delivering the INTENT vaping prevention materials (select all that apply)		
It is important to teach pupils about vaping	40	88.9
I am concerned about vaping in schools	26	57.8
I am required to teach about vaping as part of school policy	24	53.3
I wanted to take part in this study	10	22.2
Did the INTENT vaping prevention materials change your knowledge about...		
Vaping harms		
Yes, a lot	19	42.2
Yes, somewhat	18	40.0
No	8	17.8
Quitting smoking		
Yes, a lot	9	20.0
Yes, somewhat	16	35.6
No	20	44.4
According to what you know now, after delivering the INTENT vaping prevention materials…		
Vaping is…^1^		
A lot less harmful than smoking	0	0.0
A little less harmful than smoking	13	35.1
Equally as harmful as smoking	15	40.5
A little more harmful than smoking	3	8.7
A lot more harmful than smoking	6	16.2
Don’t know	0	0.0
Vaping can help people to quit smoking ^2^		
Yes	25	67.6
No	8	21.6
Don’t know	4	10.8

^1^Among those who responded ‘yes, a lot’ or ‘yes, somewhat’ to “Did the INTENT youth vaping prevention materials change your knowledge about... - Vaping harms” (n = 37); ^2^ Among those who responded ‘yes, a lot’ or ‘yes, somewhat’ to “Did the INTENT youth vaping prevention materials change your knowledge about... - Vaping for quitting smoking” (n = 37).

**Table 3:** Teachers’ perceptions of the impact of INTENT on their pupils (N = 45).

	N	%
Pupils were engaged with the INTENT vaping prevention materials		
Yes, a lot	12	26.7
Yes, somewhat	32	71.1
No	1	2.2
Don’t know	0	0.0
INTENT vaping prevention materials changed your pupils’ opinions about vaping		
Yes, a lot	8	17.8
Yes, somewhat	32	71.1
No	5	11.1
Don’t know	0	0.0
Thinking about how the INTENT vaping prevention materials have impacted your pupils, have you observed any of the following?		
Knowledge that vaping is less harmful than smoking		
Increase	33	73.3
No change	5	11.1
Decrease	4	8.9
Don’t know	3	6.7
Knowledge that vaping can help people to quit smoking		
Increase	25	55.6
No change	14	31.1
Decrease	3	6.7
Don’t know	3	6.7
Knowledge that youth who have not smoked should not vape		
Increase	28	62.2
No change	12	26.7
Decrease	2	4.4
Don’t know	3	6.7
Vaping among pupils		
Increase	6	13.3
No change	16	35.6
Decrease	9	20.0
Don’t know	14	31.1
Smoking among pupils		
Increase	1	2.2
No change	12	26.7
Decrease	10	22.2
Don’t know	22	48.9
Feedback from pupils on the INTENT vaping prevention materials		
Yes, mostly positive	10	22.2
Yes, somewhat positive	15	33.3
Yes, both positive and negative, or neutral, feedback	13	28.9
Yes, somewhat negative	2	4.4
Yes, mostly negative	5	11.1
No feedback received	0	0.0
INTENT vaping prevention materials were appropriate in encouraging pupils to make informed choices about vaping		
Yes, mostly appropriate	24	53.3
Yes, somewhat appropriate	19	42.2
Neither appropriate nor inappropriate	1	2.2
No, somewhat inappropriate	1	2.2
No, mostly inappropriate	0	0.0
Don’t know	0	0.0

**Teacher characteristics**. Teachers’ gender, age group, local council, role within the school, teacher’s vaping and smoking, frequency of finding school pupils possessing or vapes or cigarettes, whether they thought vaping and smoking are problems in their school.

**Teachers’ experiences with INTENT.** Teachers’ rating of the content of the INTENT youth vaping prevention materials, whether session plans were adhered to, ease of preparation and delivery, overall experience of delivery, reasons for delivering the materials, prior delivery of the materials, whether INTENT had changed teachers’ knowledge about vaping harms and vaping for quitting smoking, and, if so, whether teachers (after delivering the materials) perceived vaping to be less harmful, equally harmful, or more harmful than smoking, and whether they perceived that vaping could help people to quit smoking.

**Teachers’ perceptions of the impact of INTENT on their pupils.** Feedback received by teachers from pupils about INTENT, whether they thought their pupils were engaged with INTENT, thought that INTENT had changed their pupils’ opinions about vaping, thought that INTENT was appropriate for pupils to make informed choices about vaping, and whether teachers observed a change in pupils’ vaping behaviour, smoking behaviour, knowledge that vaping is less harmful than smoking, knowledge that vaping can help people to quit smoking, and knowledge that youth who have not smoked should not vape.

## Analyses

All analyses are descriptive. Findings are summarised and described in the following order: 1) teacher characteristics, 2) teachers’ experiences with INTENT, 3) teachers’ perceptions of the impact of INTENT on their pupils. An anonymised version of the dataset is available online osf.io/kprxc [31].

## Results

### Teacher characteristics

A total of 45 teachers completed the survey from schools in the following areas: in England, North-East Lincolnshire (n = 22), Cheshire and Merseyside (n = 15), Somerset (n = 6), and, in Scotland, Tayside (n = 2) (Table S1, in S1 File).

Table 1 shows the characteristics of the teachers that took part in this study. Most teachers were women (60%), had never tried vaping (64%) but had tried smoking (51%), and all except one (who preferred not to disclose their age) were between the ages of 25 and 54. About half of teachers found their pupils possessing or using vapes at least once a week (51%) and most perceived vaping as a problem in their school (96%) while corresponding estimates for cigarettes were 4% and 35%, respectively.

### Teachers’ experiences with INTENT

Table 2 shows teachers’ experiences with the INTENT vaping prevention materials. Most teachers reported that their overall experience of delivering the materials was very or somewhat positive (96%), that they liked (38%) or were indifferent to (53%) the content, adhered to the session plan all (49%) or most (42%) of the time, and found preparation and delivery easy (87% and 93%, respectively). When asked about their reasons for delivering the materials, participants could select multiple responses, and the most selected reason was that it is important to teach pupils about vaping (89%), followed by being concerned about vaping in schools (58%), and the requirement to teach about vaping as part of school policy (53%).

Most reported that the INTENT vaping prevention materials changed their knowledge about vaping harms (82%) and vaping for quitting smoking (56%). Of those who reported that their knowledge changed, most reported that they now thought that vaping can help people quit smoking (68%). However, findings for harm perceptions were mixed, with 35% accurately perceiving vaping as less harmful than smoking, 25% inaccurately perceiving vaping as more harmful than smoking, and 40% inaccurately perceiving vaping and smoking as equally harmful.

### Teachers’ perceptions of the impact of INTENT on their pupils

Table 3 shows teachers’ perceptions of the impact of the INTENT vaping prevention materials on their pupils. The majority of teachers reported that their pupils were engaged with the materials (98%) and that the materials encouraged pupils to make informed choices about vaping (96%). Most also reported that the materials changed their pupils’ opinions about vaping: specifically, that the materials increased pupils’ knowledge that vaping is less harmful than smoking (73%), that vaping can help people quit smoking (56%), and that youth who have not smoked should not vape (62%). Around a third perceived that the INTENT vaping materials did not change their pupils’ vaping (36%) or smoking (27%) behaviours and between a third and half did not know if there was a change (31% and 49%, respectively), although some perceived an increase in vaping (13.3%) and smoking (2.2%). When teachers were asked whether they received any feedback on the materials, several reported receiving mostly or somewhat positive feedback from pupils (56%).

## Discussion

The aim of this study was to assess teachers’ experiences with delivering youth vaping prevention materials to school pupils. A total of 45 teachers in England and Scotland who delivered INTENT’s vaping prevention materials took part in the study. The teachers provided information about their own perceptions and experiences with vaping and the materials delivered as well as their perceptions of their pupils’ responses. To our knowledge this was the first study to assess teachers’ experiences with vaping and delivering vaping prevention materials in schools outside of the US.

Overall, teachers reported frequently finding their pupils possessing or using vapes and perceived vaping to be a problem in schools; both to a greater extent than cigarettes/smoking. Teachers had overall very or somewhat positive experiences with INTENT’s vaping prevention materials, suggesting it may be feasible to implement in schools, and they thought that their pupils had at least somewhat positive experiences as well. They mostly felt that the materials had changed both their own knowledge and their pupils’ knowledge about vaping harms and vaping for smoking cessation, and that they mostly encouraged their pupils to make informed choices about vaping.

While the aim of INTENT’s vaping prevention materials was to reduce use and improve knowledge among *school pupils*, it is of added benefit that *teachers* perceived the materials to improve their own knowledge. These results are broadly consistent with prior research in the US finding that vaping educational materials are effective in changing teachers’ perceptions of vaping [14]. Prior interventions evaluated in the US focused on communicating to teachers the absolute harms from vaping (which are not substantiated by evidence, e.g., vaping causes heart disease, cancers) [14] whereas importantly, INTENT provided nuanced, evidence-based information about vaping and smoking risks.

Although most teachers that were surveyed reported a change in perceptions after delivering INTENT, misperceptions that vaping is equally or more harmful than smoking remained high. Importantly, our study did not collect data prior to delivering INTENT, so a pre-/post-evaluation was not possible; harm perceptions may have been higher prior to delivering INTENT, particularly since vaping misperceptions are pervasive among teachers [28] and the general population [32] in Great Britain. Pre-/post- studies (ideally RCTs, which are the gold-standard for evaluating interventions) would be required to assess changes in both teachers’ and pupils’ knowledge about vaping and smoking, as well as changes in behaviour following INTENT’s vaping prevention materials, as has been done for INTENT’s smoking prevention materials [22]. Additionally, interventions are needed across a wider range of formats and settings (e.g., mass media campaigns [3], information on labels/packaging [3], information in pharmacies and clinical settings [33]) to improve knowledge among the general population.

Findings clearly demonstrate teachers’ interest in youth vaping prevention, consistent with qualitative work in the US [29] and anecdotal reports in the UK. Most teachers delivered INTENT’s vaping prevention materials because they felt it was important to teach pupils about vaping. This may be because the majority of teachers in this study perceived vaping to be problematic in schools. Indeed, over half also reported delivering INTENT because they were concerned about vaping in schools or were required to teach about vaping as part of school policy. Teachers are critical in the provision of health education in schools and therefore shaping lifelong health behaviours [25,26]. It is already illegal to sell cigarettes and vaping products to youth in the UK and there are few other evidence-based tools available to help prevent youth vaping; teachers are therefore essential in this mission because they educate numerous young people each year and are influential in informing young people’s views. Teachers’ extensive reach, combined with the standardised nature of their role, positions them well to deliver evidence-based interventions consistently and at scale [25–27]. The teachers that were surveyed reported high rates of finding pupils possessing or using vapes and perceiving that vaping is a problem, both to a greater extent than cigarettes. This is consistent with population estimates that more children have vaped than smoked since 2022 [1] and with qualitative work in the US finding that educators perceive vapes to be easily accessible/obtainable within schools [29]. However, given that smoking prevalence remains at 5.4% among 11–17-year-olds in Great Britain [1], and cigarettes are much more harmful than vapes [2–6], it is important that vaping prevention materials are delivered alongside smoking prevention materials, as INTENT does. School policies and prevention materials for vaping and smoking should also be consistent with evidence on the risk profiles of these products (e.g., tighter smoking policies than vaping policies) to avoid giving mixed signals to school pupils; this is particularly important when teachers perceive vaping as more problematic than smoking.

This study has important strengths. It was the first study of its kind to assess teachers’ experiences of delivering vaping prevention materials in the UK. The teachers in our survey had first-hand experience of delivering vaping prevention materials in schools and so findings offer unique insights for school-based vaping interventions and the feasibility of INTENT. However, there are several limitations. First, the sample of 45 teachers was small, limited to a few areas in England and Scotland that delivered INTENT and where consent was obtained from commissioning organisations to contact schools, and not nationally representative. Findings may also not generalise to other countries, particularly because the materials delivered were UK-specific (e.g., information about legal limits of nicotine in vapes). Second, the data were from one cross-sectional survey administered to teachers after delivering vaping prevention materials, and so rely on retrospective information about the impact of the materials and teachers’ perceptions of how they were received by school pupils. The INTENT *smoking* prevention materials were effective in reducing youth smoking initiation from 36% to 29% in a pragmatic RCT conducted among school pupils; similar methods are needed to formally evaluate INTENT’s *vaping* prevention materials. Qualitative research could also be beneficial to understand teachers’ and pupils’ responses to the materials in greater depth. Third, findings may be subject to social desirability biases since this study was conducted by King’s College London in collaboration with Evidence to Impact (who design and own the licence to deliver INTENT); indeed, some teachers reported delivering INTENT specifically because they wanted to take part in this study. Fourth, while teachers were asked to confirm that they had delivered at least Session Plans 1 and 2 (sessions with the most information about the health harms of vaping) to be eligible, and over half delivered all four session plans, this was not formally monitored and many teachers did not fully complete the course.

To conclude, this study found that there were pervasive misperceptions that vaping is equally or more harmful than smoking among a sample of teachers in England and Scotland, and that these teachers reported being more concerned about vaping than smoking in schools. INTENT shows potential to challenge vaping misperceptions and facilitate school pupils to make informed choices about vaping and smoking behaviours. While further studies are required to assess the impact of INTENT on school pupils’ vaping and smoking perceptions and behaviours, this study is an important starting point among a key population.

## Supporting information

S1 File Session plan details, Table S1, Table S2. (DOCX)
