# Supplementary material for: Teachers’ experiences of delivering youth vaping prevention materials in schools in England and Scotland: A cross-sectional online survey
Source: PLoS One. 2025 Nov 7;20(11):e0335474. doi: 10.1371/journal.pone.0335474 (PMC12594386; doi:10.1371/journal.pone.0335474)
Supplement: S1 File — (DOCX) [file pone.0335474.s001.docx]

**Details of the session plans and quiz**

Details of the session plans and quiz are shown below. For the purposes of copyright, all branding has been removed.

**Session Plan 1** asked pupils to discuss what they know about vaping, including advertising, impacts of vaping (environmental, health, financial), how vaping is similar or different from smoking, and why young people try vaping. Examples of the content of Session Plan 1 are shown below.


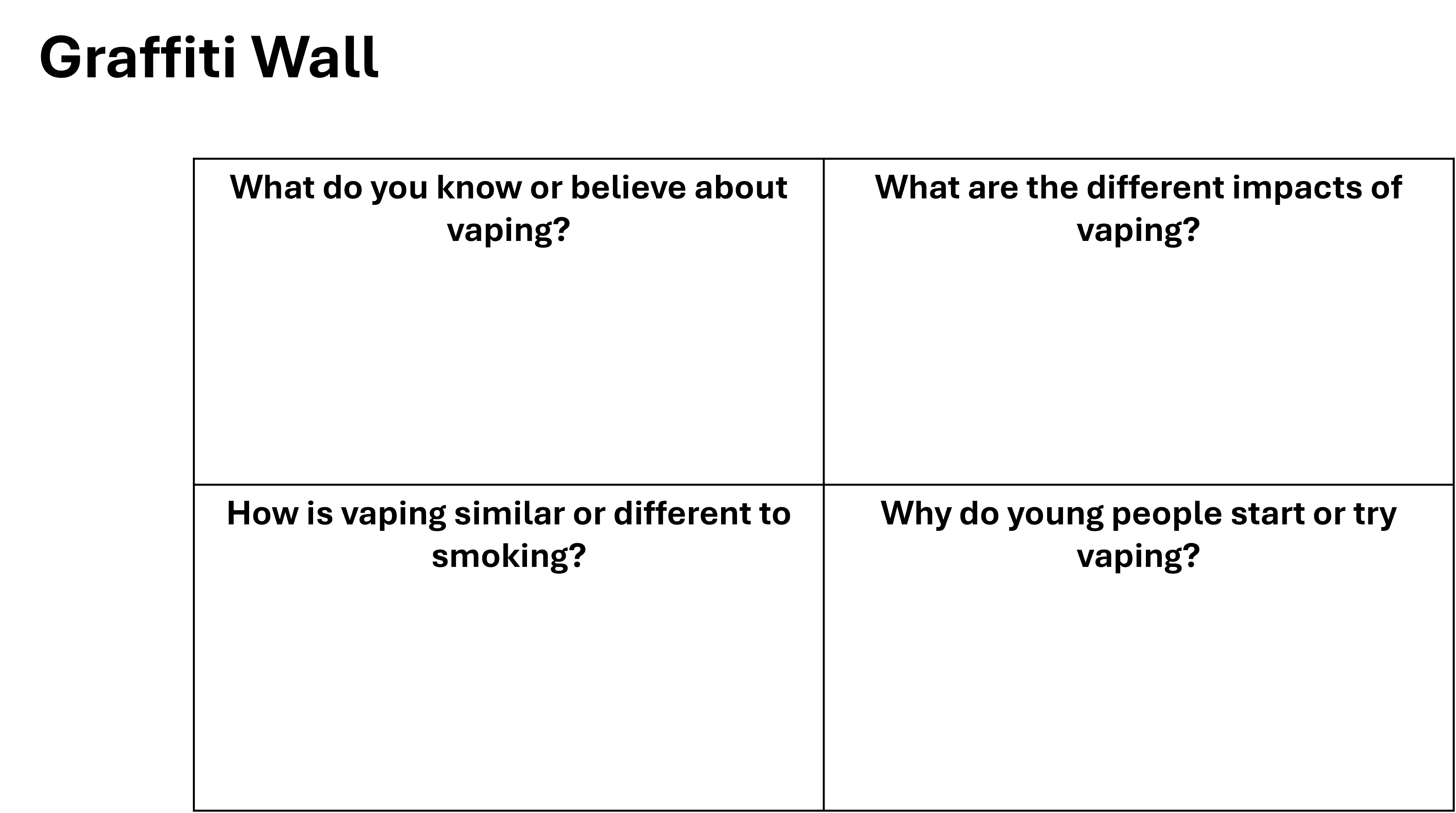


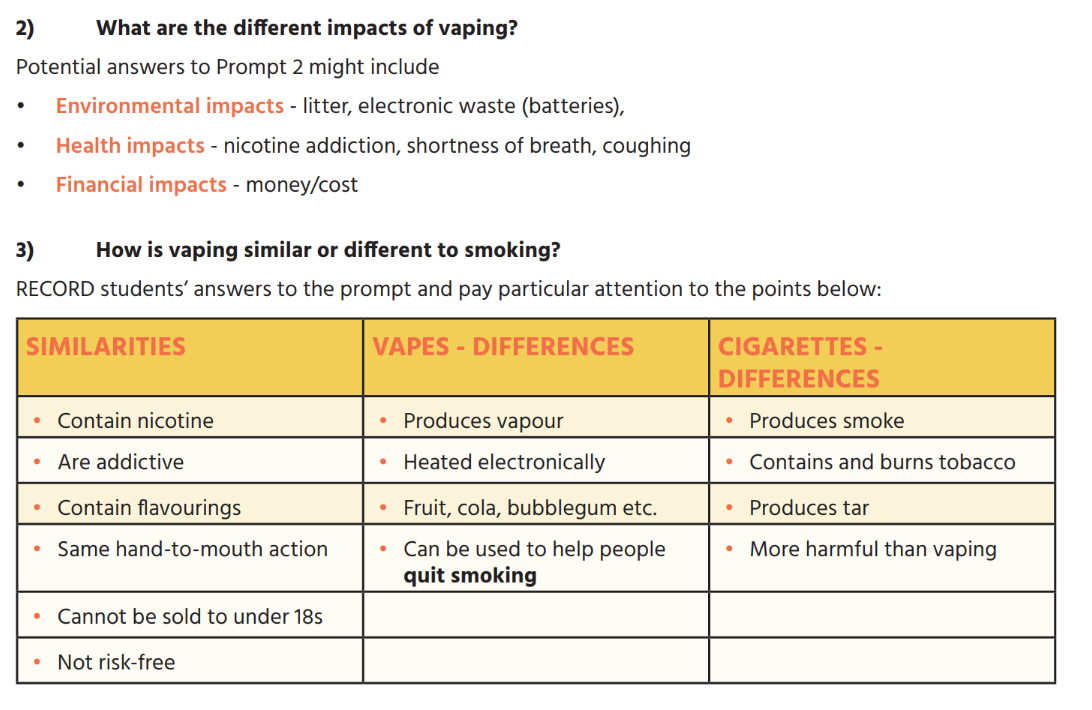


**Session Plan 2** involved pupils thinking about social influences on their health behaviour (including vaping) followed by a quiz with nine questions to test their knowledge about vaping including vaping harms. An example of the content of Session Plan 2 is shown below.


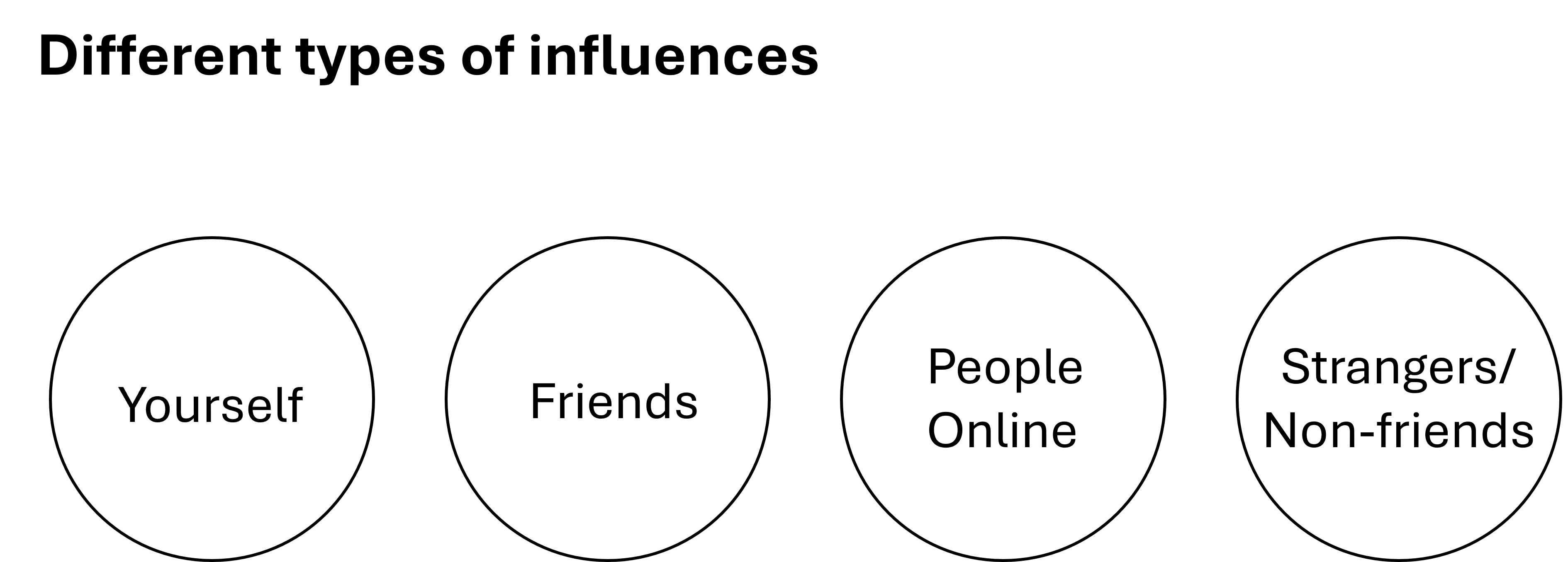


Examples of questions and correct answers in the quiz are:

1. What is the legal age of sale of vaping products? (correct answer: 18 years old)
2. Do you think vaping is more or less harmful, compared to smoking? (correct answer: less harmful)
3. Can vaping help people quit smoking? (correct answer: yes)
4. Is vaping risk-free (correct answer: no)
5. What are the risks of long-term vaping (correct answer: unknown)

As part of the quiz, pupils were provided with feedback on all answers to facilitate learning, regardless of whether answers were correct or not. One example of the feedback provided is “Heating e-liquid in a vape creates vapour, which is much less harmful than smoke created from burning tobacco. However, vaping is not harm-free! You should only use a vape if you are trying to quit smoking.”

**Session Plan 3** involved asking pupils to discuss the environmental impacts of vaping, considering what is inside a vape, different types of waste generated from vapes, as well as the responsibility of retailers in disposing of vapes. Examples of the content of Session Plan 3 are shown below.


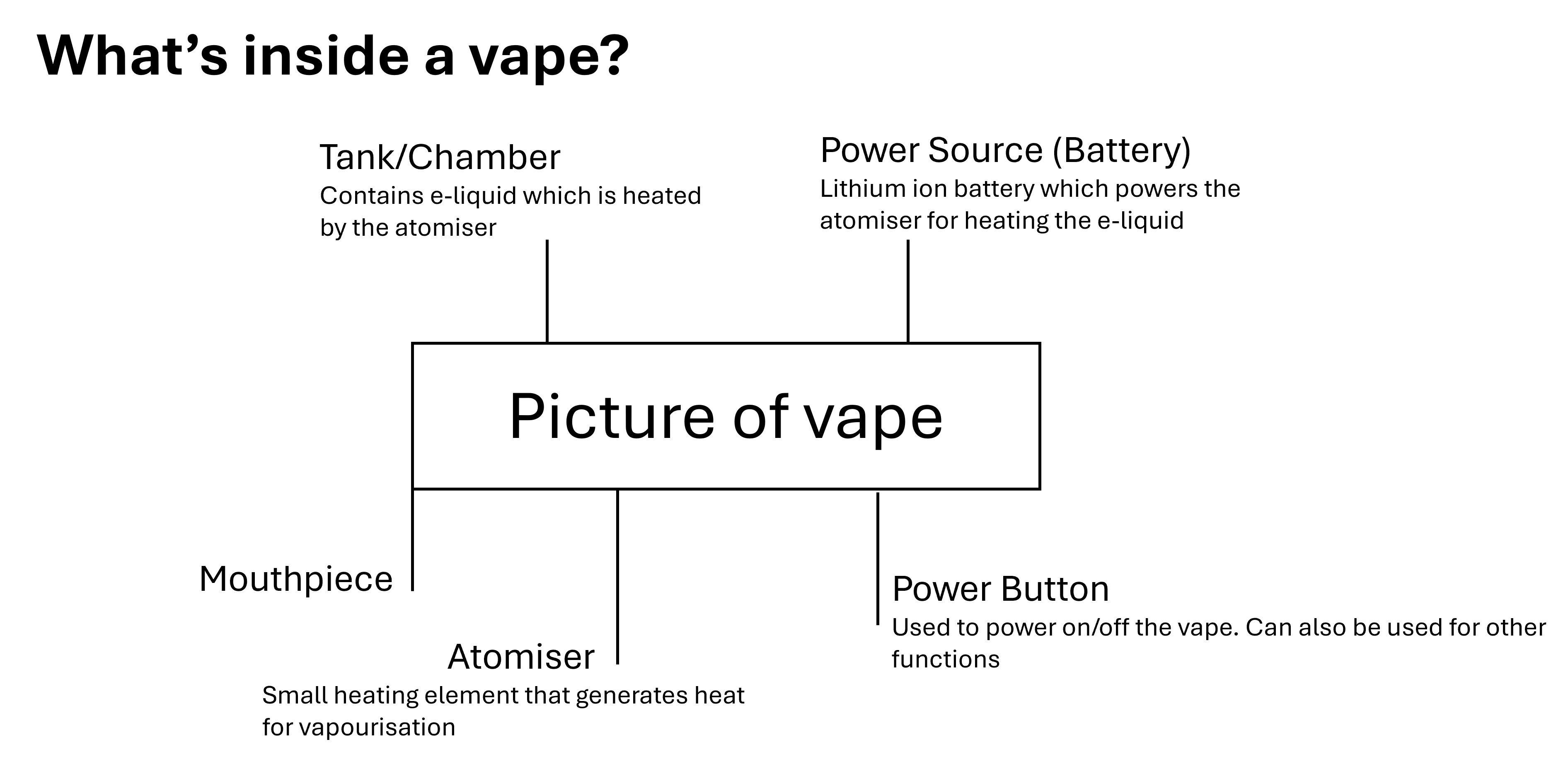


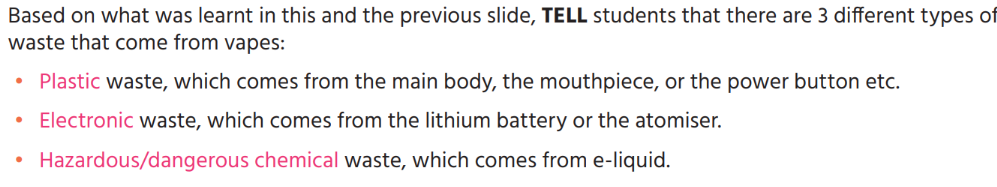


**Session Plan 4** asked pupils to consider addiction (in general, and specific to nicotine) including symptoms and withdrawal, and provided information about nicotine and how it is delivered to the brain. An example of the content of Session Plan 4 is shown below.


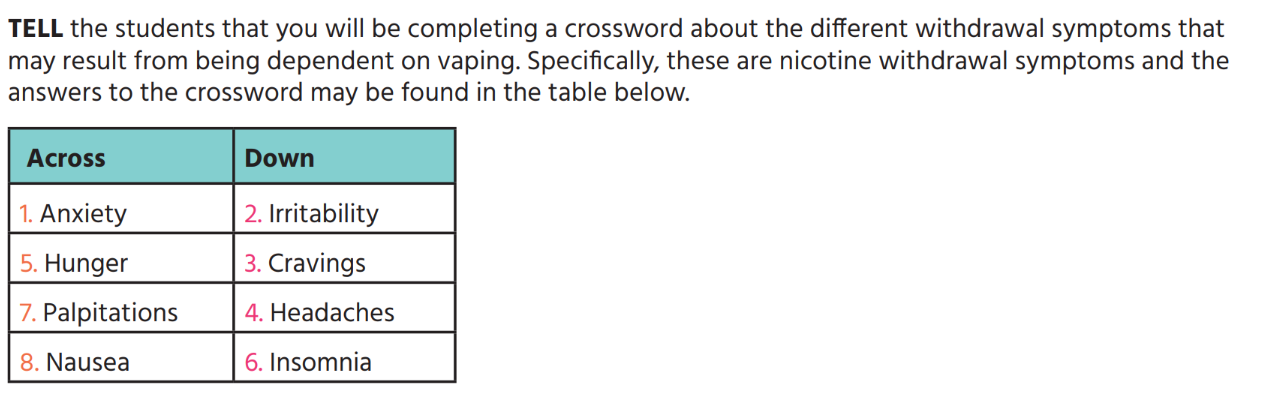


Session 4 also included a quiz with six questions to test pupils’ knowledge about nicotine addiction. Examples of questions and correct answers in the quiz are:

1. What is the addictive component of vapes? (correct answer: nicotine)
2. What is the legal limit on the amount of nicotine in vapes in the UK? (correct answer: 20mg/mL)
3. Is vaping more addictive than smoking cigarettes? (correct answer: no)

Feedback was also provided after each question, for example “As with smoking, nicotine is the addictive component in vapes. That's what makes it such an effective tool for quitting smoking! It is also less harmful as it does not rely on burning tobacco as it contains no tobacco. Many people don’t realise it, but *nicotine is not the cancer-causing chemical in tobacco cigarettes.* However, it is what makes cigarettes and vapes addictive, so it is possible for people to become addicted to vaping.”

Pupils were asked at the end of each session to create a personal plan to refuse an offer to vape. Further details of the intervention are available upon reasonable request from Evidence to Impact.

**Table S1. Details of commissioning organisations that were approached and provided consent.**

| **Commissioning organisations approached** | **Outcome** | **Number of teachers that completed the survey (N=45)** |
| --- | --- | --- |
| **England** |  |  |
| NHS Cheshire and Merseyside Integrated Care Board | Consent provided | 15 |
| North-East Lincolnshire Council | Consent provided | 22 |
| Somerset Council | Consent provided | 6 |
| Nottinghamshire County Council | Consent provided | 0 |
| Worcestershire County Council | Consent provided | 0 |
| Stoke-on-Trent City Council | Did not consent | 0 |
| Nottingham City Council | Did not consent | 0 |
| **Scotland** |  |  |
| NHS Tayside Health Board | Consent provided | 2 |

**Table S2. Teacher characteristics among those who were included in the analytic sample (N=45) and those who were excluded for not meeting the inclusion criteria or completing the survey (N=25).**

|  | Included (N=45) | |  | Excluded (N=25) | | |
| --- | --- | --- | --- | --- | --- | --- |
|  | N | % |  | N | % including ‘no response’ | Valid % |
| Role within school |  |  |  |  |  |  |
| Head of Department | 4 | 8.9 |  | 3 | 12.0 | 15.8 |
| Teacher | 28 | 62.2 |  | 8 | 32.0 | 42.1 |
| Teaching Assistant | 1 | 2.2 |  | 1 | 4.0 | 5.3 |
| Other (e.g., Assistant Principal, Progress Leader, Special Education Needs Coordinator) | 11 | 24.4 |  | 7 | 28.0 | 36.8 |
| Prefer not to say | 1 | 2.2 |  | 0 | 0.0 | 0.0 |
| No response | 0 | 0.0 |  | 6 | 24.0 | - |
| Age group |  |  |  |  |  |  |
| 18 - 24 | 0 | 0.0 |  | 1 | 4.0 | 5.3 |
| 25 - 34 | 22 | 48.9 |  | 5 | 20.0 | 26.3 |
| 35 - 44 | 11 | 24.4 |  | 5 | 20.0 | 26.3 |
| 45 - 54 | 11 | 24.4 |  | 5 | 20.0 | 26.3 |
| 55 - 64 | 0 | 0.0 |  | 3 | 12.0 | 15.8 |
| Prefer not to say | 1 | 2.2 |  | 0 | 0.0 | 0.0 |
| No response | 0 | 0.0 |  | 6 | 24.0 | - |
| Gender |  |  |  |  |  |  |
| Man | 17 | 37.8 |  | 6 | 24.0 | 31.6 |
| Woman | 27 | 60.0 |  | 13 | 52.0 | 68.4 |
| Prefer not to say | 1 | 2.2 |  | 0 | 0.0 | 0.0 |
| No response | 0 | 0.0 |  | 6 | 24.0 | - |
| E-cigarette use (vaping) |  |  |  |  |  |  |
| I currently use them daily | 1 | 2.2 |  | 1 | 4.0 | 16.7 |
| I currently use them weekly but not daily | 1 | 2.2 |  | 0 | 0.0 | 0.0 |
| I currently use them monthly but not weekly | 0 | 0.0 |  | 0 | 0.0 | 0.0 |
| I have tried them but do not use them monthly | 13 | 28.9 |  | 1 | 4.0 | 16.7 |
| I have never tried them | 29 | 64.4 |  | 4 | 16.0 | 66.7 |
| Don't know | 1 | 2.2 |  | 0 | 0.0 | 0.0 |
| No response | 0 | 0.0 |  | 19 | 76.0 | - |
| Cigarette use (smoking) |  |  |  |  |  |  |
| I currently use them daily | 1 | 2.2 |  | 0 | 0.0 | 0.0 |
| I currently use them weekly but not daily | 1 | 2.2 |  | 0 | 0.0 | 0.0 |
| I currently use them monthly but not weekly | 0 | 0.0 |  | 0 | 0.0 | 0.0 |
| I have tried them but do not use them monthly | 21 | 46.7 |  | 2 | 8.0 | 33.3 |
| I have never tried them | 21 | 46.7 |  | 3 | 12.0 | 50.0 |
| Don't know | 1 | 2.2 |  | 1 | 4.0 | 16.7 |
| No response | 0 | 0.0 |  | 19 | 76.0 | - |
